# Texas State Medical Association

**Published:** 1896-02

**Authors:** P. C. Coleman

**Affiliations:** President T. S. M. A.; Colorado, Texas


### Texas State Medical Association.

The following announcement has been sent out by President Coleman:

COLORADO, TEXAS, January 31, 1896.

DEAR DOCTOR:—The Texas State Medical Association will hold its twenty-eighth annual meeting in the city of Fort Worth, April 28th to May 1st, inclusive. Every regularly educated physician in the State, who is a graduate of a regular medical college in good standing, and who conforms to the code of ethics, should be a member of the State Association. If you are not already a member, it is a duty you owe the profession and yourself to join; and may we not hope to have the pleasure of meeting you at Fort Worth?

This is not a mere formal announcement, doctor, because the constitution requires it, but it is a personal appeal to you, and we hope you will so regard it.

We would especially urge upon all gentlemen who expect to read papers in any of the sections, to have titles of their papers in the hands of the secretaries as early in March as they can. It is not necessary for you to have the papers so early, but send the titles. This is earnestly requested by our Secretary, in order that he may prepare the programme, so as to have it in your hands at an earlier date than heretofore. I would suggest it would add materially to the interest of the discussions if the authors would designate some gentleman to open the discussion on their papers.

We are justified in saying that there is more interest manifested on the part of our members, than has ever existed before. Many local and district societies have recently passed, and others will, when they meet, pass resolutions setting forth loyalty, and urging upon their members, if not already members of the State Association, to attend the Fort Worth meeting and join.

Now, doctor, the State Association comes to you with this appeal: "The object of this Association shall be to organize the regular medical profession of the State in the most efficient manner possible; to encourage a high standard of professional qualifications and ethics; to promote professional brotherhood; to labor for the advancement of State medicine, that is, of public hygiene; of medical education; of medical jurisprudence, and public institutions for the sick and infirm." Is not such an object sufficient to enlist your support?

Lay aside your responsibilities for the time, and meet with us at Fort Worth, the city of genuine and unbounded hospitality.

Very truly and fraternally yours,

P. C. COLEMAN, M. D., President T. S. M. A.

---

**Central Texas Medical Society** met at Waco January 15, ult., and elected the following officers for the next term, six months:

J. C. Shaw, of Stranger, president; J. E. Brown, of McGregor; J. M. Strayhorn, of Bartlett, and H. C. Black, of Waco, first, second and third vice-presidents in the order named. Marvin L. Graves, of Waco, secretary, and J. H. Sears, of Waco, treasurer.

**The Northwest Texas Medical Society** met recently at Greenville, and, after going through an interesting program, elected the following officers for the ensuing year:

J. C. Erwin, of McKinney, president; A. B. Gardner, Denison, vice-president; R. D. Potts, Bonham, secretary; J. B. Stinson, Sherman, treasurer. The next meeting will be held in McKinney, the 3d Tuesday in June, 1896.

